# On chip synthesis of a pH sensitive gefitinib anticancer drug nanocarrier based on chitosan/alginate natural polymers

**DOI:** 10.1038/s41598-024-51483-z

**Published:** 2024-01-08

**Authors:** Hossein Alizadeh, Mazaher Ahmadi, Omid Heydari Shayesteh

**Affiliations:** 1 Faculty of Chemistry and Petroleum Sciences, Bu-Ali Sina University, Hamedan, Iran; 2 Nutrition Health Research Center, Hamadan University of Medical Sciences, Hamadan, Iran

**Keywords:** Non-small-cell lung cancer, Materials science, Nanoscience and technology

## Abstract

In this research, using a microfluidic chip, a nanocarrier for the anticancer drug gefitinib was synthesized. Chitosan and alginate natural polymers were utilized for the synthesis of the nanocarrier. The synthesis of the nanocarrier comprises the interaction of secondary amine functional groups of gefitinib molecules with carboxylate functional groups of alginate polymer to form the primary nucleus followed by the formation of the nanocarrier through the self-assembly of chitosan and alginate polymers on a fabricated microfluidic chip. The chip was fabricated by laser engraving poly(methyl methacrylate) polymer sheets. The nanocarrier was characterized by FT-IR, DLS, SEM, and TEM techniques. The synthesized nanocarrier had a size distribution of 5.30 ± 2.60 nm and the encapsulation efficiency percent was 68.4% in the optimum conditions. The loading efficiency was calculated as 50.2 mg g^−1^ of nanocarrier. Drug release studies showed that the nanocarrier is sensitive to pH and releases more gefitinib in acidic environments. Cytotoxicity of the synthesized nanocarrier was studied on the A549 non-small cell lung cancer, and the MTT test showed that the synthesized nanocarrier has a lower IC_50_ value than the free drug. Also, the cytotoxicity studies showed that the materials used for the synthesis of nanocarrier do not show significant cytotoxicity. Compared to the previously reported method, the developed microfluidic-assisted method showed advantages such as a faster synthesis procedure and comparable encapsulation efficiency and loading capacity.

## Introduction

The synthesis of more water-soluble derivatives and the reduction of particle size are two methods that have been employed to increase medication efficacy. However, these approaches frequently have shortcomings including non-specific medication distribution due to non-specific toxicity and delivery ^1^. Using nano- and micro-formulations is an intriguing way to increase medication bioavailability. For improved attachment, drug molecules can be adsorbed or encapsulated on biocompatible or biodegradable carriers. These formulations get across biological barriers and prolong the duration of medication circulation ^2,3^. The medicine must be released by the carrier in close proximity to cells. The carrier needs to take into account the targeted cells' unique physiological state, like in the case of malignant cells. Different carriers have been designed using tumor parameters such as pH, enzyme levels, redox species concentration, and reactive oxygen species. It is possible to modify the drug carrier to respond to particular internal or external stimuli ^4^. The distinct qualities of the impacted tissues, such as temperature, pH, redox conditions, and the overexpression of certain active molecules, are known as intrinsic stimuli. Ultrasound, magnets, heat, and light are examples of external stimuli.

The pH range of the human body is broad. Blood and regions directly in touch have a pH of 7.4, the stomach has a pH of 1–2.5, and the small intestine and colon have pH values of 7.2–7.5 and 7.9–8.5, respectively ^4,5^. Furthermore, during endocytosis, eukaryotic cells create distinct vesicle-like lysosomes and endosomes. According to studies, the pH of these vesicles is lower than that of typical lysosomes and endosomes, which are 4 and 6.5, respectively ^6^. Drug delivery techniques for cancer treatment frequently include pH-sensitive polymers ^7^. Because of the accumulation of acidic substances such as lactic acid and metabolites, tumor tissues have a pH between 5 and 6.8 higher than normal tissues ^8^. Because cancer cells use forty times more glucose than healthy cells do, low-oxygen tumor tissues produce more lactic acid as a result of the breakdown of glucose. Acidic and basic functional groups or cleavable linkages are common features of pH-sensitive polymers. They can react to pH variations in drug carriers in a number of ways, including cleavable linkages, variations in hydrogel swelling, and ionic interactions. In order to create a pH-responsive carrier for cancer treatment, the carrier's composition can include pH-cleavable bonds such as acetal and hydrazone ^4^. A cross-linker or linker that has these bonds can be used for this. The major network of the structure is lost and deteriorates in response to pH variations. Using a pH-sensitive linker, an anticancer medication may be bonded to the carrier and released in reaction to pH variations in the surrounding environment. Cross-linking hydrogels composed of polymers having basic or acidic groups, such as chitosan and alginate, can be accomplished by ionic interactions. Because the product contains functional groups that respond to pH fluctuations, it is impacted by pH. The functional groups of an acidic hydrogel get protonated and lose their ionic connection with the cross-linker when it is exposed to a suitable acidic pH. Drug release would occur as the hydrogel lost its structural integrity ^4^. The most studied mechanism in hydrogels is the change in swelling degree. Basic functional groups that can be protonated at low pH, such as chitosan, are required by the hydrogel network. Because of the repulsion interactions between positively charged groups, this results in osmotic pressure and increases the hydrogel network, which influences the degree of swelling. The degree of protonation and deprotonation of functional groups that are both acidic and basic in a polymer varies with changes in pH. As a result, depending on the pH level and the kind of polymeric precursor(s), the hydrogel network either expands or contracts ^9,10^.

Of all malignancies, lung cancer has the highest death rate and the second-highest incidence rate. In 2022, there are expected to be 236,740 new cases of cancer in the US, accounting for 12.3% of all cases, and 130,180 fatalities, or 21.4% of cancer-related deaths ^11^. One cancer medication with a genetics-based approach is gefitinib. Cells with mutations in the epidermal growth factor receptor (EGFR) gene are the target of gefitinib. On cells, EGFR is a surface protein ^12^. Particularly in non-small cell lung cancer (NSCLC), EGFRs stimulate cell proliferation and growth, which accelerates the disease's progression. The EGFR signal for cell proliferation is blocked by EGFR inhibitors. The tyrosine kinase domain of EGFR is inhibited by gefitinib. The FDA authorized gefitinib as a first-line treatment for advanced lung cancer in 2015 ^13^. Although it was formerly thought to be a revolutionary medicine, using it has had serious drawbacks. The ineffectiveness of gefitinib is hampered by its low solubility, bioavailability, and absorption ^14^. Due to gefitinib’s large volume of distribution (1400 L), it is widely distributed into healthy tissues and can have unfavorable consequences such as rash, diarrhea, hepatotoxicity, and interstitial lung disease. One of the finest methods to increase solubility and absorption is through nanomedicine ^15–17^. Liposomes, chitosan, cyclodextrin, polylactide, poly(lactic-co-glycolic acid) (PLGA), solid lipid nanoparticles, nanostructured lipid carriers, and albumin nanoparticles are among the nanoparticles that have been utilized in conjunction with gefitinib to treat lung cancer ^16^. Garizo et al. used the p28 protein from azurin to create PLGA nanoparticles. Without impacting healthy cells, the study discovered that p28 enhanced the association of nanocarriers with A549 lung cancer cells and decreased their metabolic activity. Gefitinib nanoparticle delivery has been shown in an in vivo investigation on cells to decrease lung metastases and cancer burden, providing a new approach to lung cancer treatment ^18^. Gefitinib-loaded silica nanoparticles were created by Madajewski et al.^19^ to improve the treatment of lung cancer. Gefitinib-loaded nanoliposomes were recently made by Hu et al. utilizing a non-toxic, environmentally friendly solvent and film dispersion technique. Improved circulation time, avoided protein absorption and an inhibitory impact on lung cancer are all demonstrated by an in vivo investigation. An in vitro investigation shows that the medication has a sustained release impact. According to the findings, gefitinib nanoliposomes have promise for the treatment of lung cancer ^20^. Clinical candidates for gefitinib human serum albumin-based nanoparticles have demonstrated encouraging results in treating NSCLC cancer. Even at far lower dosages, they have demonstrated superior anticancer efficacy than Iressa®. This implies that they could be useful in the management of this kind of cancer ^21^.

An alternative to traditional methods for synthesizing nanomaterials is through miniaturizing the process using microfluidic channels. Microchannels provide advantages over bulk mixing and top-down approaches due to their small dimensions, adjustable particle sizes, precise control over flow parameters, and reproducibility ^22,23^. The concentration, temperature, pH, flow rate ratio (FRR), total flow rate (TFR), and residence duration of microfluidic nanoparticles all have an impact on their characteristics ^23^. The polydispersity index (PDI) and nanoparticle size are significantly impacted by FRR. Increased shear stress and a narrower precursor stream are the results of higher FRR. This results in less nanoparticle production, shorter mixing times, and shorter mixing lengths. In more acidic environments, pH gradients can be exploited to manufacture smaller lipid nanoparticles and load drugs into liposomes. Higher temperatures have the ability to create smaller nanoparticles, speed up operations, and facilitate fluid mixing. Materials including glass, silicon, polydimethylsiloxane (PDMS), lithium niobate, and cyclic olefin copolymer (COC) can be used to create microfluidic devices ^23,24^. Depending on the material, various manufacturing methods may be applied, such as hot embossing for COC, etching for glass and silicon, and soft lithography for PDMS ^24^. In 2004, Jahn et al.^25^ produced liposomes in a microfluidic hydrodynamic focusing (MHF) channel for the first time. Via the manipulation of solvent flow rates and concentrations, liposome size was regulated. On the other hand, mass transfer in microfluidics occurs via diffusion between laminar streams because of the low Reynolds number. Long mixing periods and a drop in mixing efficiency are caused by this MHF device constraint ^26^. The next stage in the development of microfluidic nanoparticle production was to produce mixing inside microchannels in order to get around the laminar flow characteristic of microfluidics. There are two ways to mix in microchannels: passive and active. Using certain geometrical designs such as staggered serpentine, herringbone, and Tesla structures, passive mixing entails producing flow disruptions ^27^. High flow rates may be achieved with ease of setup and fabrication of passive microchannels. However, the only way to obtain tunability is to change the flow rate. Physical fields such as electricity and acoustics are used in active mixing to exert forces on fluids that facilitate mixing. Tunability may be obtained by varying external field characteristics and flow rates. On the other hand, devices are operated at low flow rates and their manufacture is complicated ^28^.

This paper reports on the development of a microfluidic device for the synthesis of gefitinib nanocarrier. The fabricated microfluidic chip utilizes a passive mixing approach using four inlets. Gefitinib drug solution is first interacted by two flows of sodium alginate solutions then faces a flow of chitosan solution for the synthesis of gefitinib nanocarrier through the self-assembly chitosan and alginate natural polymers. To our knowledge, this is the first report on the microfluidics-assisted synthesis of gefitinib nanocarrier. It is worth mentioning that the microfluidic chip was only utilized for the synthesis of gefitinib nanocarrier. The synthesized nanocarriers were then purified, characterized, and their release behavior and cytotoxicity were evaluated individually.

## Experimental

### Materials and apparatus

All reagents and chemicals have analytical purity and were obtained from Merck Company (Darmstadt, Germany), except for sodium alginate (MFCD00081310) and chitosan (average molecular weight: 50,000–190,000 Da), which were obtained from Sigma-Aldrich Company (St. Louis, MO, USA). Deionized water was used throughout this research. The gefitinib stock solution (4.47 mmol L^−1^) was prepared in 0.8% v/v acetic acid aqueous solution. The stock solution of chitosan (1.0% w/v) was prepared in 1% v/v acetic acid aqueous solution. The stock solution of 1.0% w/v sodium alginate was prepared in deionized water. 0.25 mol L^−1^ phosphate buffer was used to adjust the solutions’ pH. A 3-(4,5-dimethyl-2-thiazolyl)-2,5-diphenyl-2-H-tetrazolium bromide (MTT) assay to evaluate the cytotoxicity effect of the nanocarrier on A549 NSCLC cells was performed in the School of Pharmacy at Hamadan University of Medical Sciences, Hamadan, Iran. The UV–Vis spectra were recorded using a single beam spectrophotometer WPA model Lightwave II utilizing a quartz cell with a path length of 1 cm. A scale with an accuracy of four decimal places was used to weigh the chemical compounds and a 40 kHz ultrasonic cleaner water bath (RoHS, Korea) was used to mix and dissolve the chemicals. In order to separate the solid phase from the liquid, a centrifuge (Hettich ROTOFIX 32A) with a 15 mL centrifuge tube was used. A dynamic light scattering (DLS) analyzer model Malvern, Zetasizer NanoZ with a quartz cell (path length of 1 cm) was used to measure the size distribution of the synthesized nanoparticles. A 100 W CO_2_ infrared laser device (Rotec RT6040, Iran) was used for the construction of microfluidic chips. The solutions were pumped using a peristaltic pump (Ismatec MPC). Perkin-Elmer FT-IR (Spectrum GX) was used to record the Fourier-transform infrared (FT-IR) spectra. FESEM Tescan Mira3 was used to record field emission scanning electron microscopy (FE-SEM) images. Also, transmission electron microscopy (TEM) images were recorded with a Philips CM-120 instrument. For the TEM analysis, the samples were stained using uranyl acetate.

### Chip fabrication

**Figure 1:**
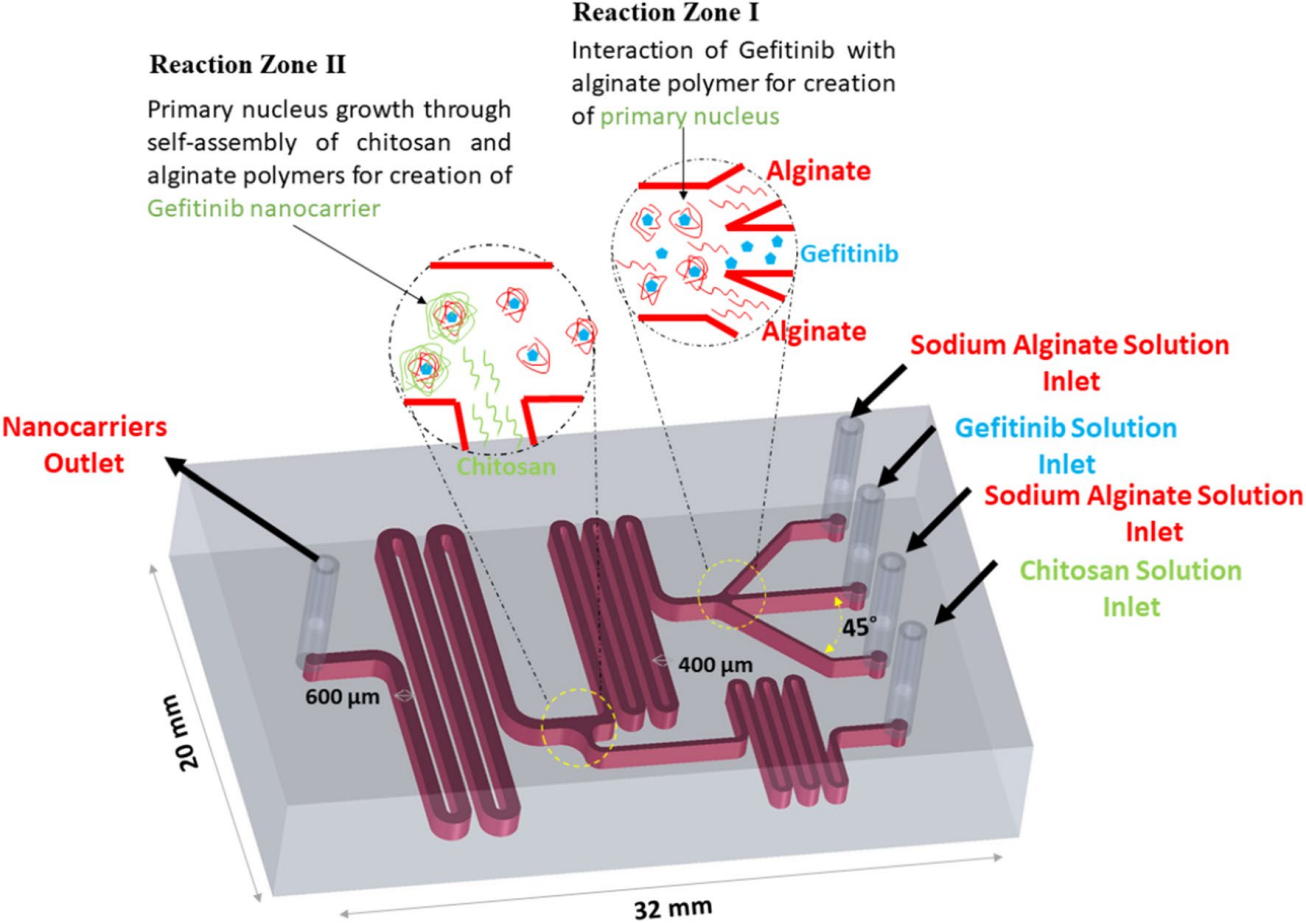
3D illustration of designed microfluidic chip for the synthesis of gefitinib nanocarrier.

SolidWorks 2021 software was used to design the microfluidic chip. The design had four inlets, two were for the sodium alginate solution, one was for the gefitinib solution, and one was for the chitosan solution (Fig. 1). After passing the desired solutions through the microchannels, an outlet was installed at the end of the chip, where the nanocarriers are collected. The diameter of each inlet channel was considered to be 200 µm (except for sodium alginate inlets that were 100 µm) and increased to 400 and 600 µm after connecting the channels. The diameter of the channels is considered in such a way that solutions with higher viscosity than water do not clog the channels and the flow created by the peristaltic pump is not reversed. Transparent poly(methyl methacrylate) (PMMA) sheets with a diameter of 2.7 mm were used to fabricate the microfluidic chip. PMMA was chosen due to its low cost, laser engravability (PDMS and normal glass do not present such characteristic), and its chemical stability under the condition used for the synthesis of gefitinib nanocarrier (over other thermoplastics). The PMMA sheets were engraved (power: 33%, speed: 500 mm s^−1^, interval: 2.5 µm) and cut according to the design using the laser device. Then, the microchannels were washed using 1-propanol in the ultrasonic bath and rinsed with deionized water. Two cut PMMA sheet pieces, one with a microchannel, inlet, and outlet, and the other without cutting were glued together using heat. In the inlet and outlet locations, the syringe head was attached using glue for connection to the peristaltic pump.

### Gefitinib-loaded nanocarrier synthesis

For the synthesis of the gefitinib nanocarrier, chitosan solution (0.20% w/v), sodium alginate solutions (0.13% w/v), and gefitinib solution (1.117 mmol L^−1^) were simultaneously pumped into the fabricated microfluidic chip (Fig. 1) at a flow rate of 2.15 mL min^−1^. The outlet solution was collected and was centrifuged at 4000 rpm for 20 min. Then, the supernatant was analyzed using the UV–Vis absorbance instrument for the evaluation of unloaded gefitinib (calibration cure equation: A_330nm_ = 0.0151C_Gef(µM)_ + 0.00099, R^2^ = 0.9952). After removing the supernatant, the synthesized nanocarriers were washed with deionized water three times. The washed nanocarriers were dried under a vacuum at room temperature and weighed. The encapsulation efficiency (EE%) and loading capacity (LC) were calculated according to Eqs. (1) and (2), respectively.

1 $$Encapsulation \;Efficiency \left( {EE\% } \right) = \left( {Unloaded \;Gefitinib/Total\; Gefitinib \;Added} \right) \times 100$$

2 $$Loading\; Capacity \left( {LC} \right)\; = \;Total \;Loaded \;Gefitinib \left( {mg} \right)/Total \;Nanocarrier \;weight\; \left( g \right)$$

The calculated values for the EE% and LC were 68.4 ± 0.6% and 50.2 ± 3.4 mg g^−1^. The proposed mechanism for the synthesis of nanocarrier comprises the interaction of secondary amine functional groups of gefitinib molecules with carboxylate functional groups of alginate polymer to form the nucleus in the reaction zone I (Fig. 1) followed by the formation of the nanocarrier through the self-assembly of chitosan and alginate polymers in the reaction zone II. The drug-free nanocarriers were synthesized using the same procedure except for replacing the gefitinib solution with deionized water.

### In-vitro release studies

To investigate the effect of pH on the drug release process, amounts of 6.4 and 5.6 mg of synthesized nanocarriers were weighed, ground, and placed inside dialysis bags (Cutoff: 15,000 kDa). The dialysis bags were placed in 10 mL of phosphate buffer with pH values of 5.5 and 7.4 in two individual falcon tubes, respectively. The tubes were placed in a shaker machine and incubated at 37 °C (50 rpm) for 120 h. At predefined time intervals, 2 mL of the solution was obtained for UV–Vis determination of the released drug. The concentration of the drug in the release medium was measured spectrophotometrically by calculation of its absorbance at 300 nm at the linear range of 2.25–11.17 μmol L^−1^.

### Cytotoxicity evaluation using MTT assay

A549 cells were seeded into a 96-well plate and cultured for 24 h in an incubator under a 5% carbon dioxide atmosphere at 37 °C to reach a cell density of 5,000 cells per well. Then, different concentrations of gefitinib nanocarrier, drug-free nanocarrier, and gefitinib were added to the wells. It should be noted that the first row as the control row contained only the culture medium (control group). The cells were incubated for 48 and 72 h. After the incubation time, 10 µL of 5% MTT solution was added to each well, and after 2–4 h of re-incubation at 37 °C (to form the formazan salt), the supernatant was discarded and 100 µL DMSO solvent was added to each well to dissolve purple crystals. By stirring at a speed of 70 rpm for 30 min, the sediments were completely dissolved, and finally, their absorbance was read at a wavelength of 570 nm using an ELISA device. Semi-logarithmic method and GraphPad 6.2.1 software were used to calculate the IC_50_ dose for the gefitinib nanocarrier and gefitinib drug in the A549 cell line. In this method, first, the logarithmic graph of the doses against the cell viability compared to the control cells is drawn by the software, and then the IC_50_ value is calculated by the software using the equation of the graph.

## Results and discussions

### Nanocarrier characterization

The synthesized nanocarrier was characterized using FT-IR, DLS, FE-SEM, and TEM techniques. First, the loading of the drug was confirmed using FT-IR spectroscopy. The nanocarrier size distribution was analyzed using DLS. The size and morphology of the nanocarriers were investigated using FE-SEM and TEM microscopy techniques.

**Figure 2:**
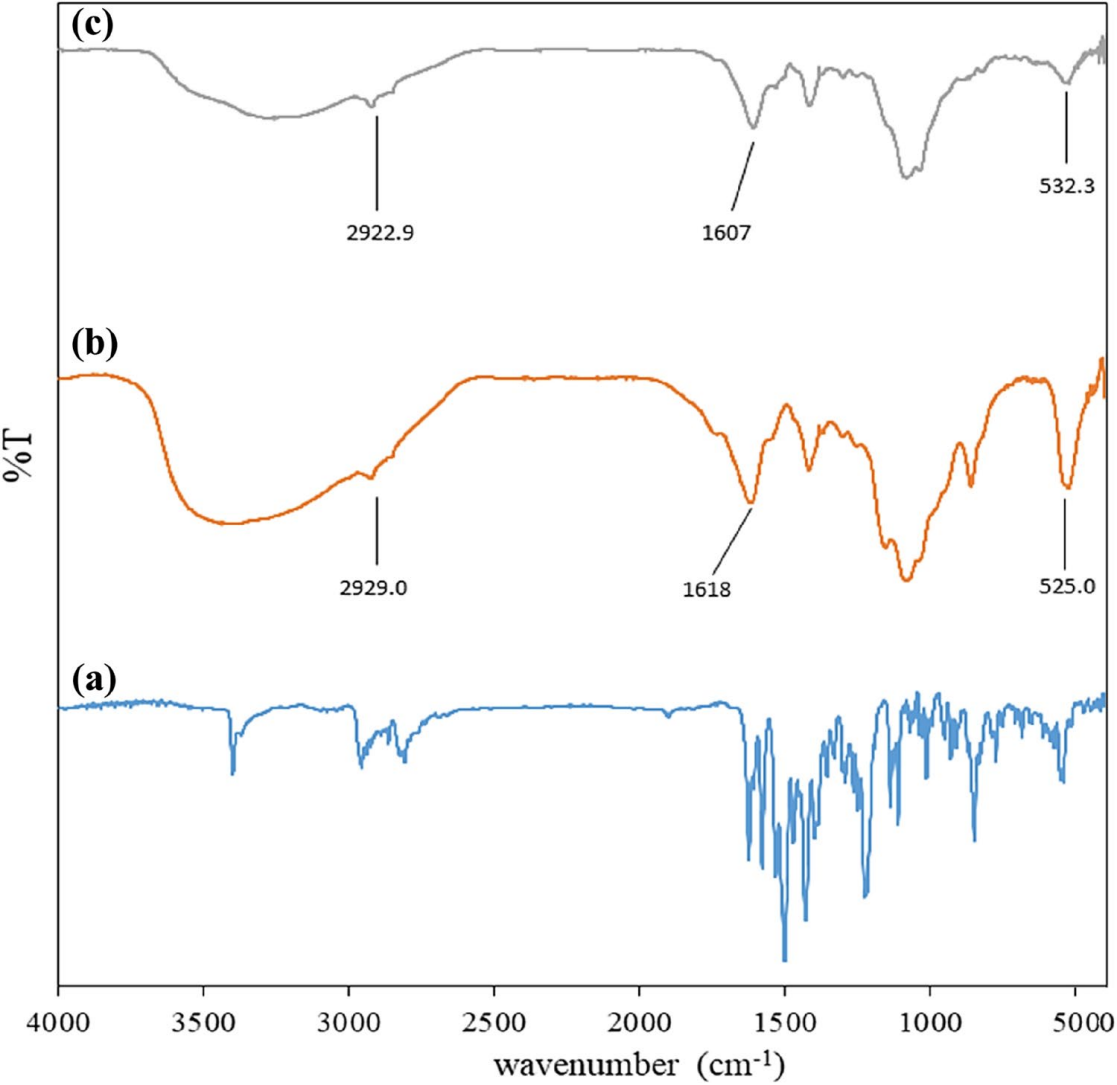
The FT-IR spectra of pure gefitinib (**a**), drug-free nanocarriers (**b**), and gefitinib-loaded nanocarriers (**c**).

**Figure 3:**
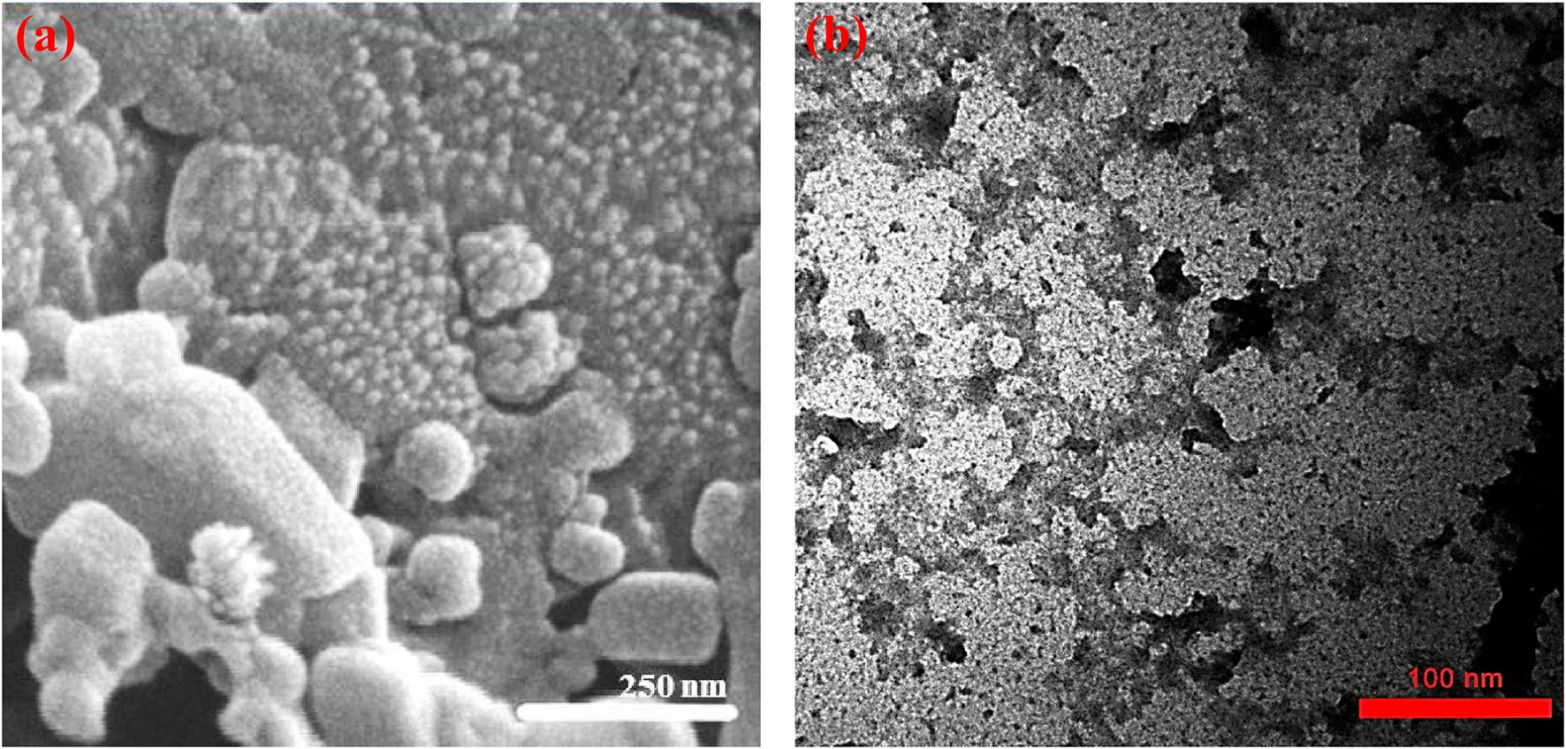
The FE-SEM (**a**) and TEM (**b**) images of the synthesized gefitinib-loaded nanocarriers.

The FT-IR spectra of pure gefitinib, drug-free nanocarriers, and gefitinib-loaded nanocarriers are presented in Fig. 2. By comparing the spectra Fig. 2b and c, it can be seen that with the addition of gefitinib, the comparative reduction of the peak intensities around of 2920 cm^−1^ (O–H in carboxylic acid groups) indicates the formation of the hydrogen bond of gefitinib with alginate. The peak in around 1600 cm^−1^ is related to the C=O bond of carboxylic acids. By comparing the spectra Fig. 2b and c, it can be seen that the frequency has decreased, which again indicates the existence of a hydrogen bond between gefitinib and alginate. In general, this change in the intensity of the peaks and the change in the frequency of the peaks indicate the formation of a hydrogen bond between alginate and gefitinib. The DLS analysis of the synthesized nanocarrier showed a size distribution of 362 ± 184 nm (PDI: 0.37) for the nanocarrier synthesized using an initial concentration of 134.10 µmol L^−1^. The FE-SEM and TEM images of the gefitinib-loaded nanocarriers are presented in Fig. 3. The images were analyzed using Digimizer software to calculate the average size of the nanocarrier. From the FE-SEM image (Fig. 3a) the average size of the nanocarrier was 13.60 ± 3.60 nm while using the TEM images (Fig. 3b) the corresponding value was 5.30 ± 2.60 nm. The significant difference in the DLS sizes and TEM/FE-SEM sizes can be attributed to the hydrogel swelling in the solution compared to the dried hydrogel. It is worth mentioning the reason for higher agglomeration state of nanoparticles in FE-SEM image compared to TEM image is different sample preparation procedures used. For TEM, the particles are fist dispersed in volatile solvent using sonication. Then, the mixture is drop-casted on the TEM grid. So, it can be expected that in TEM images, the degree of particles agglomeration would be lower than FE-SEM where the powder particles are analyzed.

### Primary optimization

**Figure 4:**
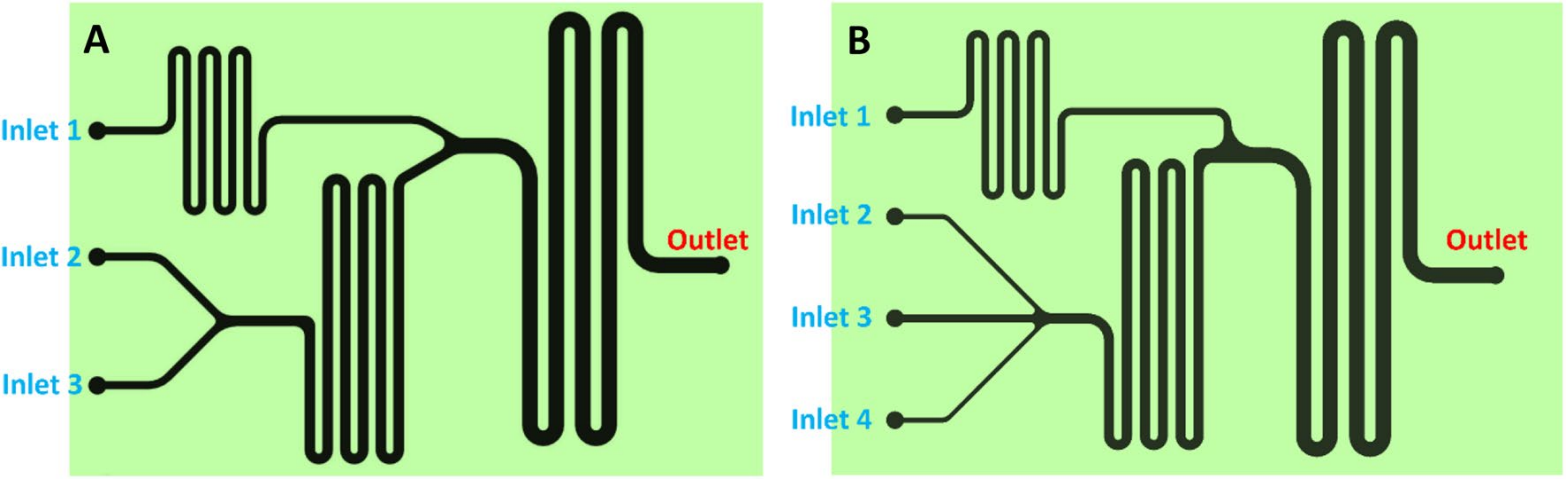
The microfluidic chip designs utilized for the synthesis of gefitinib-loaded nanocarriers.

In this section, in order to obtain particles with the smallest and narrowest size distribution, various conditions that potentially could affect the size of the nanocarrier were optimized. A primary microfluidic chip design (Fig. 4A) was utilized in this section. The chip includes three inlets and one outlet. Inlets no.1 and no.2 were the chitosan solution and sodium alginate solution entries, respectively, in all experiments of primary optimization. The inlet no.3 was the entry of deionized water in section “Chitosan to alginate concentration ratio and their concentrations” or for gefitinib solution in section “Gefitinib concentration”. To study the effect of each parameter, the DLS size and PDI values of the outlet solutions were recorded and the decision on the choice of the optimum conditions was made based on the recorded DLS data in the primary optimization experiments.

#### Chitosan to alginate concentration ratio and their concentrations

To optimize the concentration ratio of chitosan to alginate, 2 ml of the alginate stock solution (1.0% w/v) and filled up to the mark with deionized water in a 10 ml volumetric flask to obtain a concentration of 0.2% w/v. Different amounts were taken from chitosan stock solution (1.0% w/v 1%) and different concentrations were made. Table 1 shows the concentration ratio and different concentrations of chitosan and alginate.

**Table 1:** The effect of chitosan to sodium alginate concentration ratio on the DLS size of the nanocarrier (flow rate: 1.54 mL min^−1^).

Experiment No.	Sodium alginate concentration (% w/v)	Chitosan concentration (% w/v)	Chitosan to alginate concentration ratio	Peak size (nm)	SD (nm)	PDI
1	0.20	0.15	0.75	549	66	0.91
2	0.20	0.20	1.00	544	223	0.39
3	0.20	0.30	1.50	499	300	0.37
4	0.20	0.40	2.00	483	293	0.41

The obtained result from the DLS analysis (Table 1) showed that as the concentration ratio of chitosan to alginate increases, the peak size of the nanocarriers decreases. The obtained results are reasonable since by increasing the chitosan concentration and consequently, the concentration ratio, the self-assembly reaction between chitosan and alginate is prompted ^29^. It is worth mentioning that higher concentrations of chitosan caused clogging of the microchannels when combined with sodium alginate solution. Among the studied concentration ratios, the concentration ratio of 2.00 resulted in the smallest peak size. However, the PDI value for the concentration ratio of 1.50 was lower than the ratio of 2.00 meaning it had a more uniform size distribution. Thus, the concentration ratio of 1.50 was chosen as the optimum value.

To optimize the concentration of chitosan and alginate, different alginate and chitosan concentrations were prepared from the stock solutions but the concentration ratio was kept at 1.50 (Table 2). Higher concentrations than sample experiment no. 1 caused clogging of microchannels and were not studied. In low concentrations due to low concentrations (experiment no. 4), the self-assembly reaction is not such intense that causes distinct particle formation ^29^. By increasing the concentrations, the self-assembly reaction is promoted and peak sizes decrease. However, at very high concentrations, the agglomeration of particles is plausible and therefore, the DLS particle size increases. The obtained results (Table 2) showed that the optimum concentration of chitosan and alginate were 0.20 and 0.13% w/v, respectively, the smallest and most uniform particles.

**Table 2:** The effect of chitosan to sodium alginate concentration on the DLS size of the nanocarrier (chitosan/alginate ration: 1.5, flow rate: 1.54 mL min^−1^).

Experiment No.	Sodium alginate concentration (% w/v)	Chitosan concentration (% w/v)	Peak size (nm)	SD (nm)	PDI
1	0.20	0.30	492	287	0.31
2	0.13	0.20	353	194	0.34
3	0.08	0.12	436	290	0.38
4	0.07	0.10	1053	311	0.61

#### Gefitinib concentration

Different drug concentrations were prepared from the stock solution of gefitinib (Table 3). Chitosan solution with a concentration of 0.20% w/v from inlet no. 1, sodium alginate with a concentration of 0.13% w/v from inlet no. 2, and different concentrations of gefitinib from inlet no. 3 were pumped into the microfluidic chip and the outlet was analyzed using the DLS instrument (Fig. 4A). The results showed that as the concentration of gefitinib increases, the peak size decreases. When the concentration of gefitinib increases, the number of alginate-gefitinib nuclei increases and this nucleation reduces the size of the nanocarriers. According to the trend shown in Table 3, it can be expected that the size of nanocarriers will decrease with the increase in the concentration of gefitinib.

**Table 3:** The effect of gefitinib concentration on the DLS size of the nanocarrier (chitosan/alginate ration: 1.5, flow rate: 1.54 mL min^−1^, the concentration of chitosan and alginate were 0.20 and 0.13% w/v, respectively).

Gefitinib concentration (µmol L^−1^)	Peak size (nm)	SD (nm)	PDI
44.70	532	190	0.42
67.05	475	120	0.40
111.75	400	71	0.42
134.10	362	184	0.37

### Drug-loading optimization

After primary optimization experiments, the working parameters were optimized to achieve maximum EE%. In this section, the outlet of the chip was collected in a beaker containing 2 mL phosphate buffer of pH 7.4. Then, the nanocarriers were separated from the mixture using centrifugation at 4000 rpm for 20 min. The absorbance of the supernatant was recorded using the UV–Vis spectrophotometer for the calculation of the un-loaded drug and subsequently the EE%.

#### Flow rate optimization

**Figure 5:**
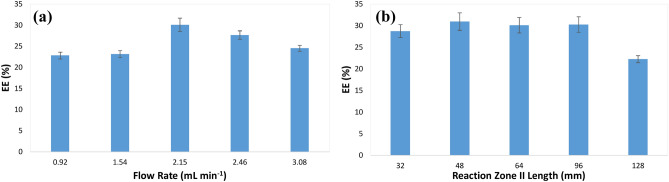
Effect of flow rate (**a**) and reaction zone II length (**b**) on the encapsulation of gefitinib using the fabricated microfluidic chip (conditions: chitosan concentration, 0.20% w/v; alginate concentration, 0.13% w/v; gefitinib concentration, 111 µmol L^−1^; reaction zone II length for evaluation of the effect of flow rate, 64 mm; flow rate for evaluation of the effect of reaction zone II length, 2.15 mL min^−1^).

In order to optimize the flow rate, chitosan with a concentration of 0.20% w/v, alginate with a concentration of 0.13% w/v, and gefitinib with a concentration of 111 µmol L^−1^ was pumped into the microfluidic chip (Fig. 4A) at different flow rates. Then, the EE% was calculated as a function of the flow rate. The results (Fig. 5a) showed that at low flow rates, the EE% is low. By increasing the flow rate, the EE% first increases and then decreases. The flow rate can affect both the mixing of chitosan-gefitinib-alginate flows and the self-assembly reaction between them. For maximum EE%, the flows should be effectively mixed and there should be enough time for the self-assembly reaction. Furthermore, at a high flow rate, there is not enough time for the interaction of gefitinib molecules with alginate polymers at the reaction zone I. Therefore, the obtained results are reasonable and the flow rate of 2.15 mL min^−1^ with an EE% of 30.10% was chosen as the optimum.

#### Reaction zone II length optimization

For the optimization of the effect of reaction zone II length, chitosan with a concentration of 0.20% w/v, alginate with a concentration of 0.13% w/v, and gefitinib with a concentration of 111 µmol L^−1^ were pumped into microfluidic chips of different reaction zone II length (Fig. 4A) at 2.15 mL min^−1^ flow rate. Then, the EE% was calculated as a function of the reaction zone II length. The results (Fig. 5b) showed that the length does not affect the EE% significantly, although the least length of 32 mm is necessary for the self-assembly reaction to occur. At high reaction zone II length, the EE% decreases probably due to the nanocarriers sticking to the wall of the microchannels as further increasing the length (> 128 mm) caused blockage of the microchannels in the microfluidic chip. Therefore, the reaction zone II length of 64 mm with an EE% of 30.11% was chosen as the optimum value.

#### Chip design optimization

In order to achieve the maximum EE%, three designs were tested under the optimized conditions. In the first design, the chip includes three inlets (Fig. 4A): Inlet no.1 for chitosan solution, inlet no. 2 for sodium alginate solution, and inlet no.3 for the gefitinib solution entry. In the second design, the same chip as the first design was utilized except for inlets no.2 and no.3 were the entries for the pre-incubated gefitinib-sodium alginate solutions (magnetic stirring for 40 min) in the third design, the chip includes four inlets (Fig. 4B): Inlet no.1 for the chitosan solution, inlets no. 2 and no. 4 for the sodium alginate solution, and inlet no.3 for the gefitinib solution entry. The results showed that design no. 2 provides better EE% (i.e., 42.0%) than design no.1 (i.e., 30.1%) due to the more effective interaction of gefitinib molecules with alginate polymers. However, the design no. 3 provided even higher EE% (i.e., 45.3%) avowing the long pre-incubation time needed for design no. 2. The observed results can be attributed to the more effective interaction of gefitinib molecules with alginate polymers in design no. 3 inside the microchannels. Therefore, design no. 3 (Fig. 4B) was utilized in the future experiments.

#### Gefitinib concentration optimization

**Figure 6:**
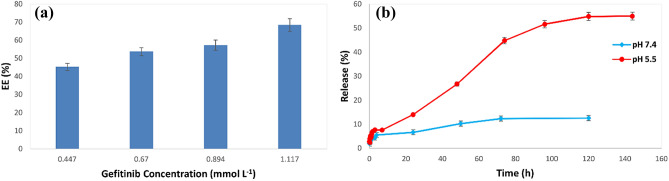
The effect of gefitinib concentration on the EE% (**a**) and the effect of pH on the release percent of the drug (**b**) (conditions: Design no. 3 (Fig. 4B); chitosan concentration, 0.20% w/v; alginate concentration, 0.13% w/v; reaction zone II length, 64 mm; flow rate, 2.15 mL min^−1^).

To study the effect of gefitinib solution concentration on the EE%, different concentrations of the drug were prepared from the stock solution and were utilized for the synthesis of gefitinib nanocarrier under the optimum conditions. The results (Fig. 6a) showed that with the increase in the concentration of gefitinib, the EE% increases. The EE% reached 68.4% at 1.117 mmol L^−1^, which was chosen as the optimal concentration. The loading capacity (LC) under this condition was 50.2 ± 3.4 mg g^−1^. Concentrations higher than this value caused microchannels to clog and were not studied. The obtained results are reasonable since increasing the drug concentration leads to the formation of a higher amount of gefitinib-alginate nucleus. It is worth mentioning that the reason for the long release experiments follow-up is to evaluate the time that the release percentage reach a constant value and thus monitoring the maximum release percentage under the experimental condition.

### In vitro drug release studies

Figure 6b shows the release experiment results at two pH values (pH 7.4 to mimic normal physiological pH and pH 5.5 to mimic tumor environment pH). It was observed that at pH 5.5 compared to pH 7.4, the release percent was higher, so the nanocarrier release behavior is sensitive to pH. Also, the drug release speed was relatively slow ensuring prolonged release of the drug. The release kinetics was studied using different kinetic mathematical models ^30^ using the Nonlinear Least Squares Regression (Curve Fitter) program, and the value of correlation coefficient (R^2^) and Root Mean Square (RMS) were obtained. The results are shown in Table 4. Korsmeier-Peppas is used to describe drug release from a polymer system by considering non-physical mechanisms. This model is useful when the release mechanism is unknown or when more than one type of drug release phenomenon is involved. According to the values of R^2^ at pH 7.4, drug release follows the Korsmeier-Peppas mathematical model, according to the value of n which is less than 0.45 (Fick’s law of diffusion), which represents the systems with a diffusion pattern with impaired release. At pH 5.5, according to the values of R^2^, the drug release kinetics follows the Korsmeier-Peppas model, and its n value is between 0.45 and 1 (abnormal displacement), which is characteristic of cases where, in addition to diffusion, other mechanisms in drug release play a role^30^.

**Table 4:** Kinetic parameters obtained from fitting experimental data with different kinetic models.

Release model	Parameter	pH = 5.5	pH = 7.4
Zero-order ($C = K_{{\text{o}}} t + C_{0}$)	k˳ (h^−1^)	5.016	3.782
	R^2^	0.962	0.860
	RMS	3.869	1.445
First order $\left( {C = C_{{\text{o}}} \left( {1 - Exp\left( { - Kt} \right)} \right)} \right)$	k (h^−1^)	68.52	10.33
	R^2^	0.960	0.713
	RMS	4.026	2.106
Higuchi (M_t_/M͚ = k·t^1/2^)	k (h^−1/2^)	4.741	1.390
	R^2^	0.969	0.706
	RMS	3.417	2.163
Korsmeier-Peppas (M_t_/M͚ = k·t^n^)	k (h^−n^)	3.548	3.730
	R^2^	0.972	0.963
	RMS	3.304	0.742
	n	0.564	0.256

In the zero-order kinetic model, C is the drug concentration at time t, C_0_ is the initial concentration of the drug, t is the time, and K_0_ is the zero-order rate constant. In the first-order kinetic model, C˳ is the initial concentration of the drug and K is the first-order rate constant. In the Higuchi kinetic model, M_t_ is the amount of drug released at time t, M_∞_ is the amount released at the infinite time, and k is Higuchi's speed constant. In the Korsmeier-Peppas kinetic model, t is the time, k is the Korsmeier-Peppas release rate constant, and n is the release exponent.

### Cytotoxicity against A549 non-small lung cancer cell line

**Figure 7:**
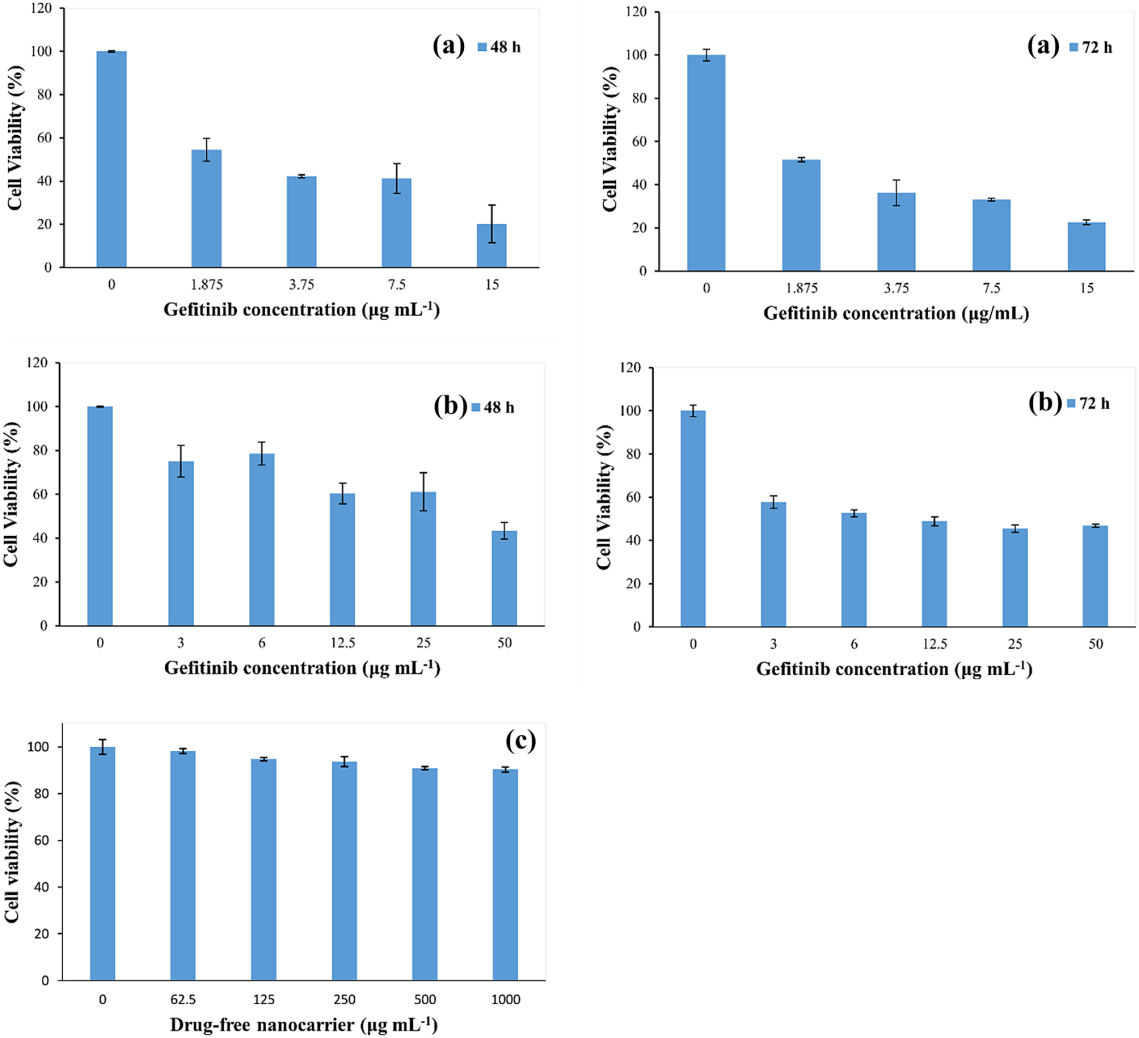
Cell viability of A549 after 24 h and 48 h incubation with different concentrations of gefitinib as gefitinib nanocarrier formulation (**a**) and pure gefitinib (**b**). Cell viability of A549 after 48 h incubation with different amounts of drug-free nanocarrier (**c**).

In this section, a comparison was made between free gefitinib drug, gefitinib nanocarrier, and drug-free nanocarrier (Fig. 7). The IC_50_ values after 48 h incubation with gefitinib and gefitinib nanocarrier were 28.090 and 3.189 µg mL^−1^, respectively. The corresponding IC_50_ values after a 72-h incubation were 12.380 and 2.721 µg mL^−1^, respectively. It was observed that the IC_50_ of gefitinib nanocarriers was lower than free gefitinib. Thus much smaller amounts of the drug are needed to kill 50% of cancer cells, which indicates the higher lethality of gefitinib nanocarriers than free gefitinib. The reason for this lethality is that the drug is released slowly and continuously and does not allow cancer cells to grow and divide within 48 and 72 h. Also, it was observed that with the increase in the dose of nanocarriers containing gefitinib, A549 cell death increased. Furthermore, according to Fig. 7c, the precursors used in the synthesis of nanocarriers showed no significant cytotoxicity. Since, the precursors used for the synthesis of the gefitinib nanocarrier (i.e., chitosan and alginate) have been already characterized for their biocompatibility and biodegradability ^31^, it can be expected that the nanocarrier would show no significant cytotoxicity toward normal cells.

## Conclusions

In this work, a microfluidic-assisted method was developed for the synthesis of gefitinib nanocarrier using chitosan and alginate natural polymers. Compared to previous similar works (Table 5), smaller particles were synthesized at a lower synthesis time even when the precursors for the nanocarrier synthesis were the same. The EE% and LC were comparable to previously developed methods for gefitinib nanocarrier synthesis. The use of microchannels for mixing and self-assembly reaction between chitosan and alginate polymers causes the interaction of substances at the micro level leading to decreased reaction time keeping high efficiency of self-assembly reaction. The sensitivity of the gefitinib nanocarrier to pH causes the release of the drug in acidic environments (pH of cancer cells) to be several times higher than in neutral environments. This feature can potentially protect healthy cells from being damaged by the gefitinib drug. The sensitivity to pH and the slow release of the gefitinib nanocarrier caused the IC_50_ value of the gefitinib nanocarrier to be decreased compared to the free drug in the A549 cell line. Also, cytotoxicity studies showed that the materials used for the synthesis of nanocarriers do not show significant cytotoxicity.

**Table 5:** A comparison of the synthesized gefitinib nanocarrier with previously developed nanocarriers.

Synthesis method	Nanocarrier main components	Synthesis time (min)	Loading capacity (mg g^−1^)	Encapsulation efficiency (%)	Drug	Cell line	Nanocarrier size (nm)	Refs.
Ionic pre-gelation alginate followed by chitosan complexation	Chitosan and alginate natural polymers	90	6.8	–	Nifedipine	–	20–50	^32^
Ionic pre-gelation alginate followed by chitosan complexation	Chitosan and alginate natural polymers	120	0.3	–	Lovastatin	–	50–80	^33^
emulsification solvent volatilization	PLGA, chitosan, and alginate polymers	300	256.7	87.23	Resveratrol	RAW 264.7 macrophages	255	^34^
Emulsion solvent evaporation	PLGA and PVA polymers	300	–	–	Gefitinib	A549	222	^35^
Ionic gelation	Chitosan, and sodium tripolyphosphate	60	–	80.7	Gefitinib	QGY	80.8	^36^
Emulsion solvent evaporation	PLGA and γ-PGA	240	–	89.5	Gefitinib	SAS	548.5	^37^
Microfluidics assisted self-assembly of alginate and chitosan	Chitosan and alginate natural polymers	5	50.2	68.4	Gefitinib	A549	5.3	This work

Poly(lactic-co-glycolic)acid (PLGA); Polyvinyl alcohol (PVA); γ-Polyglutamic acid (γ-PGA).

## Data Availability

Experimental data will be available on request, please contact the corresponding author at m.ahmadi@basu.ac.ir.
